# Navigational cue effects in Alzheimer's disease and posterior cortical atrophy

**DOI:** 10.1002/acn3.566

**Published:** 2018-04-20

**Authors:** Keir X. X. Yong, Ian D. McCarthy, Teresa Poole, Tatsuto Suzuki, Biao Yang, Amelia M. Carton, Catherine Holloway, Nikolaos Papadosifos, Derrick Boampong, Julia Langham, Catherine F. Slattery, Ross W. Paterson, Alexander J. M. Foulkes, Jonathan M. Schott, Chris Frost, Nick Tyler, Sebastian J. Crutch

**Affiliations:** ^1^ Dementia Research Centre Department of Neurodegeneration UCL Institute of Neurology University College London London United Kingdom; ^2^ Pedestrian Accessibility and Movement Environment Laboratory Department of Civil, Environmental and Geomatic Engineering Faculty of Engineering Science University College London London United Kingdom; ^3^ Department of Medical Statistics Faculty of Epidemiology and Population Health London School of Hygiene and Tropical Medicine London United Kingdom; ^4^ School of Architecture and Urban Planning Harbin Institute of Technology Shenzhen Graduate School Shenzhen China; ^5^ Oxford Health NHS Foundation Trust Oxford United Kingdom; ^6^ Department of Computer Science Faculty of Engineering Science University College London London United Kingdom

## Abstract

**Objective:**

Deficits in spatial navigation are characteristic and disabling features of typical Alzheimer's disease (tAD) and posterior cortical atrophy (PCA). Visual cues have been proposed to mitigate such deficits; however, there is currently little empirical evidence for their use.

**Methods:**

The effect of visual cues on visually guided navigation was assessed within a simplified real‐world setting in individuals with tAD (*n* = 10), PCA (*n* = 8), and healthy controls (*n* = 12). In a repeated‐measures design comprising 36 trials, participants walked to a visible target destination (an open door within a built environment), with or without the presence of an obstacle. Contrast and motion‐based cues were evaluated; both aimed to facilitate performance by applying perceptual changes to target destinations without carrying explicit information. The primary outcome was completion time; secondary outcomes were measures of fixation position and walking path directness during consecutive task phases, determined using mobile eyetracking and motion capture methods.

**Results:**

Results illustrate marked deficits in patients’ navigational ability, with patient groups taking an estimated two to three times longer to reach target destinations than controls and exhibiting tortuous walking paths. There were no significant differences between tAD and PCA task performance. Overall, patients took less time to reach target destinations under cue conditions (contrast‐cue: 11.8%; 95% CI: [2.5, 20.3]) and were more likely initially to fixate on targets.

**Interpretation:**

The study evaluated navigation to destinations within a real‐world environment. There is evidence that introducing perceptual changes to the environment may improve patients’ navigational ability.

## Introduction

Dementia‐related visual impairment is often overlooked in Alzheimer's disease (AD), possibly due to vision frequently being equated to visual acuity, usually normal in AD,^1^ or patients being less likely to report visual dysfunction.^2^ However, typical AD (tAD) patients with normal ophthalmological examination often demonstrate impairments in corticovisual function,^3^ consistent with pathological involvement of parietal and temporo‐parietal regions.^4^ While corticovisual dysfunction may manifest in visual processing deficits in early stage tAD,^5^ it is a defining feature of posterior cortical atrophy (PCA), a neurodegenerative syndrome characterized by early, progressive visual impairment with relative preservation of episodic memory, usually underpinned by AD pathology.^6^, ^7^, ^8^ PCA patients exhibit various visual deficits including markedly impaired visuospatial ability, restrictions in the effective visual field and excessive visual crowding.^9^, ^10^ Such deficits contribute towards environmental disorientation, a core clinical feature of PCA.^7^, ^11^

The visual environment's impact on people with AD has previously been emphasized, with patients disproportionately relying on conspicuous landmarks for navigation.^12^, ^13^ Such reliance may arise from a combination of diminished capacity to generate, access or maintain map‐like spatial representations, and differential impairment of corticovisual functions. In tAD, processes involved in landmark recognition may be spared relative to those particularly supporting spatial mapping.^14^ In tAD and PCA, a restricted window of spatial attention may limit optic flow perception and promote the role of object‐based cues for guiding orientation.^15^, ^16^ In PCA, eye fixation position may be especially influenced by visually salient features that are conspicuous due to low‐level perceptual factors (e.g., contrast).^17^, ^18^ Such findings invite investigation of whether particular landmarks and object‐based cues support navigation in both tAD and PCA patients demonstrating a relative sparing of object relative to spatial processing and representation.

Previous studies suggest contrast‐ and color‐based cues assist bathroom‐finding and reduce wandering for people with dementia,^19^, ^20^, ^21^ although evidence is limited.^22^, ^23^ Motion‐based cues are promising, given how aspects of visual motion detection may be relatively preserved in tAD and PCA.^24^, ^25^, ^26^ The current investigation intends to maximize the translational potential of findings through assessing cue effects across different patient phenotypes and environmental conditions within a controlled real‐world setting. Quantitative exploration of navigation is enabled through concurrent tracking of physical location and gait.^27^

Our main hypothesis was that contrast‐ and motion‐based visual cues would facilitate visually guided navigation for both tAD and PCA patients. Our subsidiary hypothesis was that navigation would be less efficient in PCA relative to tAD, given the greater extent of corticovisual impairment. The primary outcome was an overall measure of navigational performance: time taken to reach a target destination. Secondary outcomes were intended to explore mechanisms through which cues might support navigation during successive phases of visually locating and walking to the target. During the initial search phase, cues were anticipated to increase the likelihood of target fixation in patients, while minimizing the proportion of time spent fixating targets before initiating the walking phase. During the subsequent walking phase, cues were anticipated to increase the directness of routes to destinations.

## Methods

### Participants

Ten tAD patients (mean age: 66.2 ± 5.0, range: 59–74; male/female: 4/6; height [cm]: 168.4 ± 11.4, Mini‐Mental State Examination [MMSE]: 18.6 ± 4.9), 8 PCA patients (mean age: 64.1 ± 6.1, range: 57–75; male/female: 4/4; height [cm]: 169.0 ± 7.0; MMSE: 19.8 ± 45.4), and 12 healthy controls (mean age: 63.7 ± 4.1, range: 58–72; male/female: 6/6; height [cm]: 169.5 ± 12.1) were enrolled. PCA patients fulfilled clinical criteria for PCA^7^, ^11^ and contemporary research criteria for probable AD.^28^ tAD patients fulfilled research criteria for a diagnosis of typical amnestic AD.^28^ All groups were of comparable age, gender, and height, and patient groups were of comparable disease severity based on MMSE score. Participants did not report a history of ophthalmological conditions, and retinal imaging excluded life or sight limiting changes in 5/10 tAD and 8/8 PCA patients. Molecular pathology (18F amyloid imaging performed as part of another investigation or CSF) was available for 5/10 tAD patients and 4/8 PCA; all were consistent with AD pathology (positive amyloid scan on standard visual rating or CSF A*β*_1‐42_ ≤450 and/or tau/A*β* ratio >1). Prior ethical approval for the study was provided by the National Research Ethics Service Committee London Queen Square and written informed consent obtained from all participants.

### Background neuropsychology

**Figure 1:**
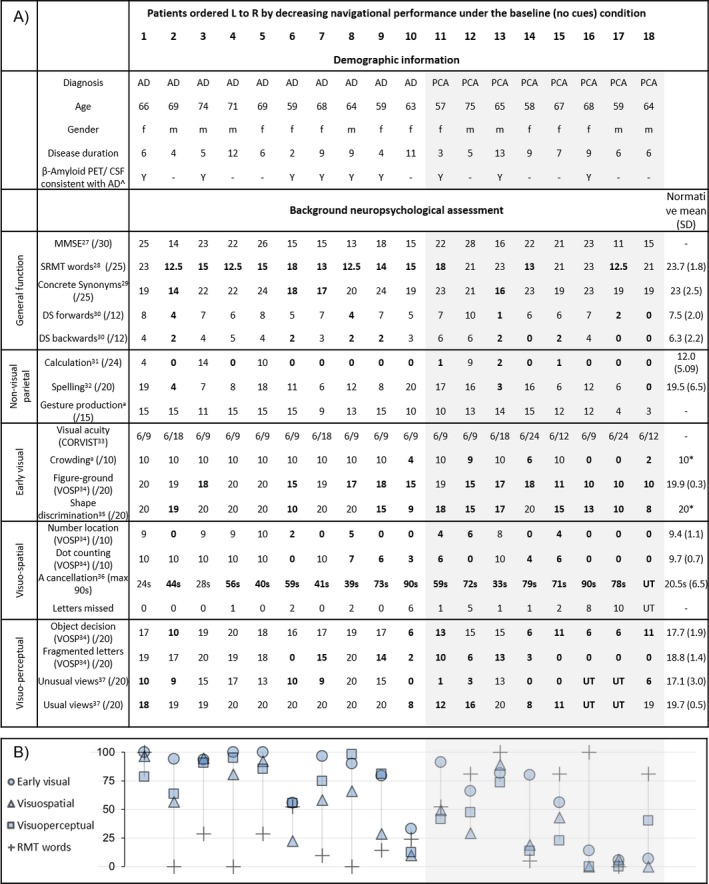
(A) Patient demographic information and background neuropsychological assessment; (B) Composite scores for visual processing domains and SRMT performance. The PCA group was more impaired on composite scores (Wilcoxon rank‐sum: Early: *z* = −2.32, *P* = 0.021; Visuoperceptual: *z* = −2.85, *P* = 0.004; Visuospatial: *z* = −2.05, *P* = 0.041), with weak evidence of greater impairment on the SRMT in the tAD group (*z* = −1.71, *P* = 0.088). Visual acuity but not contrast sensitivity was assessed; there was weak evidence for poorer acuity in the PCA than the tAD group (LogMar equivalent: *z* = 1.73, *P* = 0.085). Patients are arranged left to right in order of navigational performance from those taking the least to the most time to complete under the baseline (no cues) condition. Impaired scores (performance below 5th%ile) are highlighted in bold font. Mini‐Mental State Examination^41^; Short Recognition Memory Test^42^; Concrete synonyms^43^; Digit span forwards/backwards^44^; Graded difficulty arithmetic^45^; Graded difficulty spelling test^46^; Cortical Visual Screening Test^47^; Visual Object and Space Perception Battery^48^; Oblong edge ratio 1:1.20^49^; Letter Cancellation^50^; Usual/Unusual Views^51^; ^a^Unpublished. *Healthy controls do not make errors. SRMT, short recognition memory test; PCA, posterior cortical atrophy; tAD, typical Alzheimer's disease.

Neuropsychological tests were administered to PCA and tAD patients. Tests of early visual, visuo‐perceptual, and visuo‐spatial processing (Fig. 1A) were transformed and averaged to form composite scores for each visual domain (Fig. 1B). Raw scores for visual processing tests, along with the short recognition memory test for words (SRMT), were transformed onto a standardized range: 0 (minimum achieved by any patient) − 100 (maximum).^10^

### Stimuli

**Figure 2:**
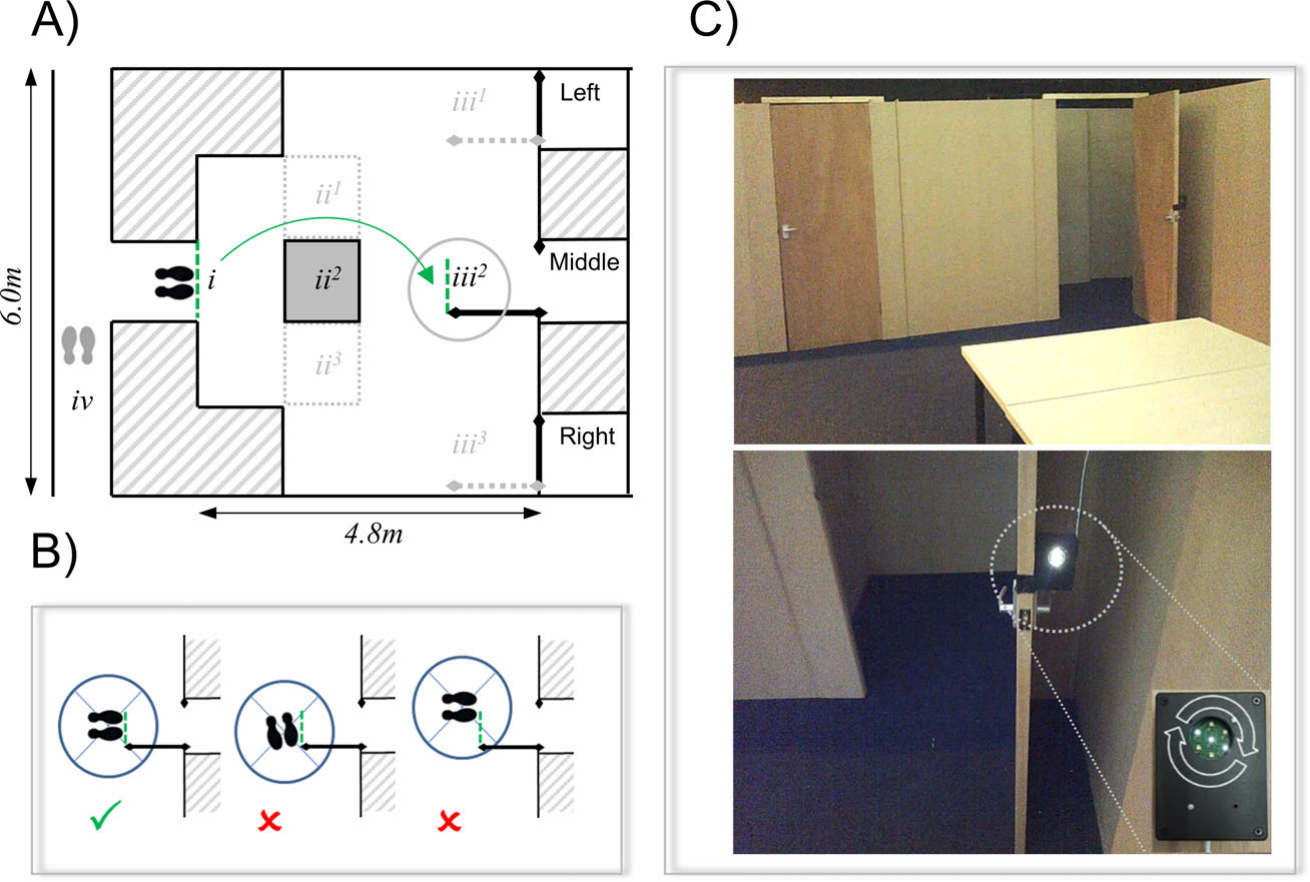
(A) i – starting position, ii^1‐3^ – obstacle positions (chosen to interrupt direct path to target destinations), iii^1‐3^ – target positions, iv – participant position between trials; (B) point defined where participants reached target; (C) right door with obstacle under CCue + motion; arrows indicate direction of motion pattern movement. The setting was constructed at a pedestrian environment laboratory (PAMELA) able to simulate real world environments in a controlled manner.

A simplified environment to assess visual cues was constructed at the Pedestrian Accessibility and Movement and Environment Laboratory (PAMELA) (Fig. 2). The setting consisted of a room (main dimensions: 6 m[W] × 4.8 m[D] × 2 m[H]) with an entry corridor serving as the trial starting point, and three doors (0.76 m[W] × 2 m[H]) separated by panels (1.64 m[W] × 2 m[H]). For each trial, one of the doors was opened at 90° indicating the target. There was high color contrast between walls and floor, consistent with design recommendations.^29^ The experiment was designed only to explicitly require spatial representation within the range of immediate perception; doors were visible from the starting point (all within 23.8° of visual angle at a distance of 4.8 m) and the task could, in principle, be completed using only visual information available at the start of each trial. Two cues were designed to promote target localization through increasing target visual salience (Fig. 2C), giving three cue conditions:

1. No cue (baseline condition).
2. Contrast‐cue (CCue): a black box (83 mm[W] × 111 mm[H] × 43 mm[D]) above the target door handle (1.10 m[H]).
3. Contrast/Motion‐cue (CCue + motion): as for 1 but also displaying a rotating white light pattern on the black box at 4 Hz and at 1800 (millicandelas) through an aperture of 34 mm diameter using seven LEDs.

For half the trials, an obstacle (1.2 m[W] × 0.7 m[H] × 1.2 m[D]) was placed between the starting point and the target so that it interrupted the most direct path to the relevant target door (Fig. 2Aii^1‐3^).

### Procedure and apparatus

At the beginning of the experiment all participants were instructed to “walk through the open door”; no reference was made to visual cues. Instructions were repeated as a prompt once per trial if participants became overtly distracted, or attempted to open a closed door. Participants began each trial with their feet centered on the starting line (Fig. 2Ai). The start time was verbally signaled by the experimenter (“Start”), preceded by a countdown from three. Participants had a maximum of 60 sec to reach the target (Fig. 2Aiii^1‐3^ and B). Between trials, participants waited outside the test room to limit their view of the experimental setting (Fig. 2Biv).

Trials were administered through a repeated‐measures design ensuring an equal number of trials for each of the following conditions: cue (Baseline, CCue, CCue + motion), target position (Left, Middle, Right), obstacle (Obstacle, No Obstacle). The three cue conditions were arranged in one of three counterbalanced variants of a Latin square design within six sets of three trials, with cue condition always changing between each trial. Two testing blocks were carried out, each block comprising all 18 possible combinations of cue/target position/obstacle, making 36 trials overall. Combinations were assigned randomly to participants to control for order effects. In this way, the experimental design was implemented to assess effects of cue rather than target position or obstacle conditions on outcomes. A mobile eyetracker (SensoMotoric Eyetracking Glasses 1) recorded gaze location at 30 Hz. Motion sensors, wireless inertial measurement units (IMUs: Xsens MT), were used to record the movement of both feet at 50 Hz, with analysis of walking paths based on left foot displacement.

### Outcome measures

#### Primary outcome: completion time

Trial time was defined as time taken from starting time to both feet reaching the target (Fig. 2B). Starting times were manually determined from each verbal “Start” using Begaze Experiment Suite 3.5; cut‐off was defined as 60 sec after “Start”. Trials were discontinued when participants: (1) did not reach the target within cut‐off; (2) walked back over the starting line before cut‐off; (3) attempted to open closed doors more than once within cut‐off. For discontinued trials, times were treated as censored at 60 sec, based on the premise that participants would be unable to reach the target within that time (see Table 1 for % of censored trials by group). Two patients (1 tAD, 1 PCA) completed only the first block of 18 trials due to time constraints; one PCA patient's second testing block was removed from analysis due to experimenter error in the order of trial presentation.

**Table 1:** Summary of data collected, trials censored at the cut‐off time of 60 sec, and medians and interquartile ranges for observed: (A) completion times; (B) proportion of trials where target was fixated; (C) for participants who did fixate, proportion of time where target was fixated during the initial period of trials (FI); (D) walking path SI

	Controls (*N* = 12)	tAD (*N* = 10)	PCA (*N* = 8)
Data available for both testing blocks (36 trials)	12	9	6
Data available for first testing block (18 trials)	0	1	2
Completion time			
Participants with trial time data for all 36 trials completed within cut‐off time of 60 sec (%)	12/12 (100%)	8/10 (80.0%)	3/8 (37.5%)
Trials completed within cut‐off time of 60 sec/Trials for which data are available (%)	432/432 (100%)	333/342 (97.4%)	224/252 (88.9%)
Completion times (within participant medians) (sec): median (25th%tile, 75th%tile) [range]	4.40 (3.66, 4.99) [2.96–5.21]	7.55 (6.26, 10.31) [4.72–20.70]	8.36 (6.46, 26.53) [5.11–51.41]
Fixation measures: whether target was fixated/FI			
Participants with fixation data for all 36 trials (%)	9/12 (75.0%)	8/10 (80.0%)	5/8 (62.5%)
Trials with fixation data/Trials for which data are available (%)	342/432 (79.2%)	306/342 (89.5%)	198/252 (78.6%)
Number (%) of trials (with fixation data) where target was fixated	198/342 (57.9%)	182/306 (59.5%)	115/198 (58.0%)
% time fixating target (FI) for trials where target was fixated (within participant medians): (median [25th%tile, 75th%tile]) [range]	19.19 (11.48, 30.61) [6.45–51.31]	19.51 (8.40, 25.61) [6.53–33.01]	15.70 (9.94, 19.51) [7.77–27.22]
SI			
Participants with SI data for all 36 trials completed within cut‐off time of 60 sec (%)	7/12 (58.3%)	7/10 (70.0%)	3/8 (37.5%)
Trials with SI data and completed within cut‐off time of 60 sec/Trials for which data are available (%)	427/432 (98.8%)	329/342 (96.2%)	222/252 (88.1%)
SI (within participant medians): (median [25th%tile, 75th%tile]) [range]	0.95 (0.95, 0.96) [0.92–0.99]	0.94 (0.91, 0.95) [0.73–0.98]	0.93 (0.76, 0.96) [0.51–0.98]

For the proportion of trials completed within cut‐off time under baseline and cue conditions, see Table S1. FI, fixation index; SI, straightness index; tAD, typical Alzheimer's disease; PCA, posterior cortical atrophy.

#### Secondary outcome measures

##### Fixation measures

Eyetracking data were analyzed during an initial time period for each trial, with the intention of assessing how participants visually searched for their destination. This fixation period was defined as starting 2000 msec before the start time and ending when a participant's feet crossed the starting line (Fig. 2Ai). Two fixation outcome measures were used: (1) a binary variable for whether or not the target was fixated (yes/no); (2) for the subset of trials where the target was fixated, fixation index (FI) was a continuous measure of the proportion of the fixation period spent fixating the target (range 0–1). Fixation position was manually categorized (Wall, Floor, Table, Door [Left, Middle, Right], Other) using Begaze Experiment Suite 3.5. Owing to the low sampling frequency of the mobile eyetracker (30 Hz), saccades could not be analyzed. Eyetracking data were excluded for five participants (1 tAD, 2 PCA, 2 control) as calibration was inadequate. Eyetracking data were not available for one block of one control participant, owing to recording error.

##### Walking path SI

Walking path data were analyzed for the time period from a participant's feet crossing the starting line to when they reached the target. IMU accelerations were converted to laboratory coordinates; velocity was calculated, corrected for sensor drift based on when feet were in contact with the ground, and integrated to estimate foot position relative to a point of origin using dead reckoning.^27^ Walking path straightness index (SI) was calculated as a ratio of the shortest possible route to the length of the route actually taken by a participant, with a range (0–1) where 1 indicated maximum straightness.^30^ SIs were unavailable for 14 trials owing to IMU recording error.

### Statistical methods

Completion times were log‐transformed and a two‐stage analysis approach adopted. In stage one, participant‐specific mean log‐transformed trial times for each cue condition were estimated, allowing for censoring of some trial times at 60 sec and for non‐constant within‐participant variability. In stage two, comparisons of these mean levels were made within and between groups, giving equal weight to each participant in each group. Performing an analysis giving equal weight to each participant, while allowing for censoring and for heteroscedasticity, would have been more complex without a two‐stage approach.

In detail: in stage one a censored normal regression model relating log‐transformed trial time to cue, door and obstacle was fitted separately for each participant (interactions were not included, since censored values for certain combinations of the predictors precluded parameter estimation for some participants). From these models, fitted mean log‐transformed trial times were computed for each of the three cue conditions (for participants without censoring these equaled the simple means of all log trial times by cue condition). In stage two, linear regression models incorporating fixed participant effects compared mean log‐transformed trial times between cue conditions within groups. In addition, overall between‐group comparison was made by calculating the mean of the three cue‐specific means and comparing these group specific means using a generalized least squares model that allowed different variances in the three groups. Results on the log‐transformed scale were back‐transformed to geometric mean trial times and reductions in geometric means.

For fixation measures, two models were used as in many trials the participant never fixated the target. The first was a mixed effect logistic regression with fixation (yes/no) as the outcome, random effects of participant, and fixed effects of group, cue, door and obstacle and the 3 two‐way interactions between group and each other variable. Marginal probabilities of fixating were estimated for each combination of group/cue condition.

The second fixation model only included trials where the target destination was fixated. FI is a continuous measure of the proportion of time spent fixating the target. We used an empirical logit transformation, which makes the distributional assumption of normality more plausible by transforming FI's (0, 1) bounded interval so that it is unbounded. After transformation, effects of cue and differences between groups are expressed as odds ratios, multiplicative factors that act on the proportion expressed as odds. For example, the proportion 0.25 (1/4) becomes 0.33 (1/3) when expressed as odds: multiplying this by an odds ratio of 2 converts the odds to (2/3), which is 0.4 expressed as a proportion. A mixed effects linear regression estimated the odds ratios, with the same fixed/random effects and interactions as the model for the binary fixation outcome. Estimated values of FI for each combination of group/cue condition were calculated by back transforming mean logit‐transformed proportions.

SI is the ratio of the shortest possible path length to the length of the path actually taken. The bounded nature of SI makes it implausible that experimental conditions have either additive or multiplicative effects on SI. For example, suppose that for a particular person a change in a particular experimental condition increases the SI from 0.5 (baseline) to 0.7. On an arithmetic scale this is a change in 0.2 and on a multiplicative scale it is a 40% increase. If we now have a second individual who has a baseline SI of 0.9 both arithmetic increase in 0.2 and a 40% increase lead to impossible values above 1. For this reason, we analyzed SI using the same empirical logit transformation as for FI. Using this logit‐transformed SI, we carried out the two‐stage modeling approach used for the completion time analyses. This allowed for variability in SI being different between participants and also for trials censored at 60 sec (which in turn censored SI). Odds ratios and estimated values of SI for each combination of group/cue condition were reported.

**Figure 3:**
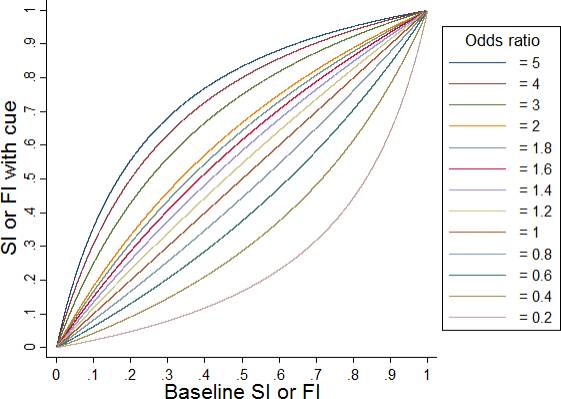
Graph illustrating selected values of the odds ratio comparing secondary outcome measures between groups and cue conditions. For example, given an estimated odds ratio of 1.2, the figure shows that if the baseline FI or SI is 0.7 then FI or SI with the cue condition is expected to be 0.74, that is the cue condition increases the proportion of initial time spent fixating on the door (for FI) or shortens the path taken by the participant (for SI), compared with baseline. FI, fixation index; SI, straightness index.

Figure 3 illustrates interpretation of FI or SI odds ratios comparing groups and cue conditions.

## Results

**Figure 4:**
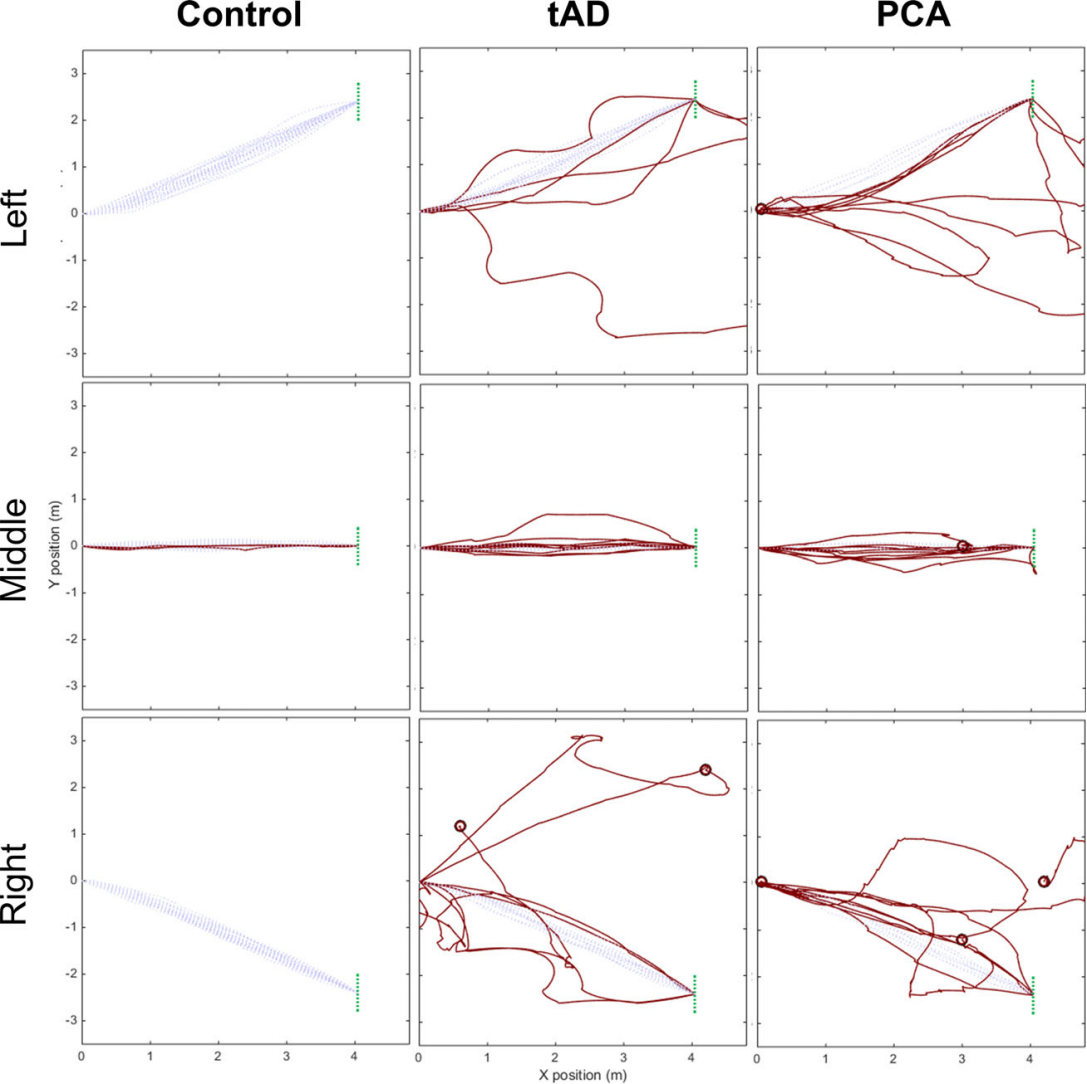
Walking paths for control, tAD and PCA groups to left, middle and right door without obstacle, under baseline condition (no cues) generated using dead reckoning. Paths were estimated using foot velocity to calculate relative displacement between each step, and so do not show absolute position.^27^ Data are presented from when participants crossed the starting line. Coloured paths are particularly indirect relative to controls (<control mean SI – 3SD). First and last data points for walking paths are corrected to reflect trial start (*x* = 0, *y* = 0) and end positions (Left: *x* = 4.04, *y* = 2.4; Middle: *x* = 4.04, *y* = 0; Right: *x* = 4.04, y = 2.4). Circles represent end positions for censored trials. tAD, typical Alzheimer's disease; PCA, posterior cortical atrophy; SI, straightness index.

Summaries of observed outcomes are in Table 1. The proportion of censored trials ranged from 2.6% in the tAD to 11.1% in the PCA group; no control trials were censored. Walking paths under baseline conditions (no cue or obstacle) are shown (Fig. 4), combining all target door positions.

### Primary outcome: completion time

Task performance was less efficient in both tAD and PCA groups relative to controls. Averaged across all conditions, patients took two to three times as long to complete trials (estimated relative completion time: tAD versus Controls: 2.1 [95% CI: 1.49, 2.96]; PCA versus Controls: 2.99 [1.66, 5.41]). There was no evidence of a difference in completion times between patient groups (PCA vs. tAD: 1.43 [0.73, 2.77]).

Table 2 shows estimated completion times, percentage changes and confidence intervals for different cue conditions and groups. There was a statistically significant estimated 11.8% reduction in mean time with CCue, relative to baseline (no cues) in patients overall. Adding motion patterns to the contrast block (CCue + motion) resulted in a smaller (and not statistically significant) 6.5% reduction in mean time. There was no evidence that the effect of CCue + motion was different from CCue alone.

**Table 2:** Estimated geometric means and percentage reduction in completion time results between cue and baseline conditions for tAD, PCA, combined patient group and controls

Primary outcome: completion time						
	Geometric mean^a^ (sec)			Percentage reduction in completion time (95% CI)		
	Baseline	CCue	CCue + motion	CCue vs. baseline	CCue + motion vs. baseline	CCue + motion vs. Ccue
tAD	9.54	8.25	8.53	13.50% (5.18, 21.09)	10.50% (1.89, 18.35)	−3.47% (−13.4, 5.61)
PCA	12.97	11.71	12.80	9.74% (−9.64, 25.69)	1.28% (−19.91, 18.73)	−9.37% (−32.8, 9.96)
Patients combined	10.93	9.64	10.22	11.85% (2.54, 20.26)	6.51% (−3.36, 15.44)	−6.05% (−17.25, 4.08)
Controls	4.16	4.16	4.18	−0.13% (−2.14, 1.84)	−0.61% (−2.63, 1.37)	−0.48% (−2.50, 1.49)

tAD, typical Alzheimer's disease; PCA, posterior cortical atrophy; CCue, Contrast‐cue; CCue + motion, Contrast/Motion‐cue.

a Geometric mean is the exponentiated mean of the estimated log transformed completion times.

Results for separate patient groups show the same direction of effect, although the estimated reduction in time taken was smaller and not statistically significant in the PCA group. There was also no evidence that the addition of motion patterns to the contrast block made a significant difference for any group. For controls, there was no evidence of differences between cue and baseline conditions. Formal tests of differences between interaction terms found no evidence that, relative to baseline, either CCue (*χ* ^2^(1)=0.15; *P* = 0.70) or CCue + motion (*χ* ^2^(1)=0.80; *P* = 0.37) had a different effect in tAD compared with PCA.

### Secondary outcome measures

Table 3 shows comparisons of secondary outcome measures between cue and baseline conditions.

**Table 3:** Estimated comparisons of secondary outcome measures between cue and baseline conditions for tAD, PCA, and combined patient groups. Comparisons are expressed as: (A) odds ratios for whether or not the target was fixated; (B) for the sub‐group of trials where fixation did take place, odds ratios for the proportion of time spent fixating the target (FI); and (C) odds ratios of walking path SI

Secondary outcomes						
(A) Target fixated						
	Estimated marginal probability of fixating			Conditional odds ratio (95% CI)		
	Baseline	CCue	CCue + motion	CCue vs. baseline	CCue + motion vs. baseline	CCue + motion vs. Ccue
tAD	0.52	0.56	0.68	1.22 (0.66, 2.28)	2.30 (1.21, 4.36)	1.88 (0.99, 3.56)
PCA	0.50	0.63	0.63	1.91 (0.86, 4.22)	1.91 (0.86, 4.22)	1.00 (0.45, 2.22)
Patients combined	0.51	0.59	0.66	1.49 (0.91, 2.44)	2.11 (1.28, 3.49)	1.42 (0.86, 2.35)
Controls	0.57	0.60	0.56	1.19 (0.67, 2.13)	0.96 (0.54, 1.70)	0.80 (0.45, 1.44)

(B) %time fixating target (FI)^a^						
	Estimated FI			Odds ratio (95% CI)		
	Baseline	CCue	CCue vs. baseline	CCue vs. baseline	CCue + motion vs. baseline	CCue + motion vs. Ccue
tAD	0.13	0.20	0.17	1.71 (1.15, 2.56)	1.44 (0.98, 2.11)	0.84 (0.58, 1.22)
PCA	0.19	0.13	0.15	0.63 (0.39, 1.04)	0.78 (0.48, 1.27)	1.23 (0.77, 1.96)
Patients combined	0.15	0.17	0.16	1.10 (0.81, 1.51)	1.09 (0.81, 1.48)	0.99 (0.74, 1.33)
Controls	0.19	0.19	0.20	0.98 (0.68, 1.40)	1.08 (0.75, 1.57)	1.11 (0.77, 1.60)

(C) Walking path SI						
	Estimated SI			Odds ratio (95% CI)		
	Baseline	CCue	CCue vs. baseline	CCue vs. baseline	CCue + motion vs. baseline	CCue + motion vs. Ccue
tAD	0.93	0.94	0.95	1.25 (0.96, 1.64)	1.37 (1.05, 1.78)	1.09 (0.83, 1.42)
PCA	0.88	0.89	0.88	1.16 (0.54, 2.50)	1.01 (0.47, 2.17)	0.86 (0.40, 1.87)
Patients combined	0.91	0.92	0.92	1.21 (0.84, 1.76)	1.19 (0.82, 1.73)	0.99 (0.68, 1.43)
Controls	0.97	0.97	0.97	1.00 (0.94, 1.07)	0.98 (0.92, 1.05)	0.98 (0.91, 1.04)

tAD, typical Alzheimer's disease; PCA, posterior cortical atrophy; FI, fixation index; SI, straightness index; CCue, Contrast‐cue; CCue + motion, contrast/motion‐cue.

a Values for this observed outcome were taken from only the subset of trials where the target was fixated.

#### Fixation measures

Averaged across all conditions, there were no significant differences between groups in whether or not the target destination was fixated (relative odds of fixation: tAD vs. Controls: 1.06 [0.41, 2.75]; PCA vs. Controls: 1.04 [0.36, 3.03]; PCA vs. tAD: 0.98 [0.33, 2.92]). Similarly, for trials where the target was fixated, there were no significant differences for the proportion of time spent fixating the target during the initial period of each trial (relative odds of FI: tAD vs. Controls: 0.86 [0.47, 1.57]; PCA vs. Controls: 0.81 [0.42, 1.60]; PCA vs. tAD: 0.95 [0.47, 1.89]).

Table 3A and B compares fixation measures for different cue conditions and groups. For the combined patient group, there was an estimated doubling of the odds of the target being fixated with CCue + motion, relative to baseline; for CCue alone the direction of effect was the same but not statistically significant. There was no evidence that the effect of CCue + motion was different from CCue.

Results for separate patient groups show the same direction of effect, but the estimated increase in odds of fixating, relative to baseline, was not statistically significant for the PCA group and only significant for tAD group with CCue + motion, the latter result providing weak evidence that adding motion patterns made some difference compared with having only the contrast block.

As shown in Table 3B for trials where the target door was fixated, for the tAD group both cue conditions were associated with an increase in the proportion of time spent fixating the target before initiating walking, although the evidence was borderline significant for CCue + motion. In contrast, a decrease in the proportion of time spent fixating the target was suggested in the PCA group, although this was borderline statistically significant for CCue and not statistically significant for CCue +motion.

Formal tests of differences between interaction terms found some evidence of a directionally different effect in the PCA group compared with the tAD group under both CCue (*χ* ^2^(1)=9.44; *P* = 0.002) and CCue + motion (*χ* ^2^(1)=3.77; *P* = 0.052) relative to baseline. Consistent with the different direction of effect in patient groups, there were no statistically significant results for the combined patient group (Table 3B). There was also no evidence that the addition of motion patterns made a significant difference in any group.

#### Walking path SI

Overall, averaged across all conditions, there was evidence that patients took less direct paths from the starting line to the target door than controls (a lower SI indicates a longer path). The estimated relative odds of SI for tAD versus controls was 0.50 (0.29, 0.84) and for PCA versus Controls was 0.24 (0.07, 0.76). There was no evidence of a difference in SI between patient groups (PCA vs. tAD: 0.47 [0.13, 1.72]).

As shown in Table 3C, directionally there was some suggestion of a benefit to directness from using cues, but this was not significant in the combined patient or PCA groups, and only borderline statistically significant in the tAD group. Once again, there was no evidence that the addition of motion patterns to the contrast block made a significant difference in any group. Formal tests of differences between interaction terms found no evidence that, relative to baseline, either CCue (*χ* ^2^(1)=0.03; *P* = 0.86) or CCue + motion (*χ* ^2^(1)=0.53; *P* = 0.47) had a different effect in the tAD group compared with the PCA group.

## Discussion

This study investigated “real‐world” navigation to visible destinations in patients with tAD and PCA, often considered the visual variant of AD, within a controlled environment. Overall, patients with PCA or tAD took on average two to three times longer to reach target destinations than controls, with motion capture data emphasizing tortuous routes taken by some patients. Some individuals were unable to complete the task within 10 times the mean controls’ completion time; others became disorientated to the point that they eventually doubled back to the starting point. Such performance may reflect significant functional navigational problems reported by many patients and carers, at least in unfamiliar settings. Our findings provide empirical evidence that a visual cue facilitates real world navigation, reducing time to destination and increasing the likelihood of patients fixating a target destination before initiating walking.

The primary outcome, completion time, provided an overall measure of participants’ navigation. While this measure is associated with age‐related factors, groups were of comparable age, gender, and height. Furthermore, cue effects were assessed from within‐participant comparisons facilitated by our use of a repeated‐measures experimental design (within‐participant comparisons being more precise than those made between participants, because each participant acts as their own control). While findings are from a small and heterogeneous group of patients, the level of evidence provided is supported by the following: the number of observations per participant, the statistical method allowing for different variances in different participants, and the randomized and counterbalanced experimental design controlling for order effects both between‐ and within‐participant.

Overall, the effect of cues on completion time exhibited two trends that also appeared broadly consistent with the secondary outcome analyses. First was evidence in the combined patient group that at least one cue condition had a beneficial effect compared to having no cue – which for completion time was an 11.8% reduction when the contrast block (CCue) was present. Second there was no evidence that the effect on completion times of adding the motion pattern (CCue + motion) was any different from the effect of CCue by itself.

The contrast block introduced features that are perceptually low‐level, yet unique within the setting. Bottom‐up visual search is driven by such features, and its integrity relative to higher‐order visual and spatial functions in these patients may partially underlie CCue effects on completion time.^31^, ^32^ However, there was an unexpected lack of evidence of a benefit of CCue + motion over CCue on primary and secondary outcomes, the only exception being weak evidence in the tAD group that the addition of motion patterns increased the number of trials where patients fixated on the target door. A greater benefit of CCue + motion had been anticipated following previous case reports of intact motion recognition in PCA.^25^, ^26^ This study's findings may indicate that the frequency of the motion pattern was too low; while motion perception may be relatively preserved in tAD, this may only be at certain frequencies.^33^ Another possibility is that patients could detect but not consciously perceive or locate motion patterns, consistent with previous studies outlining discrepancies between unremarkable ocular motor reflexes in response to moving stimuli and elevated motion perception thresholds in AD, relative to controls.^34^

The evidence of reduced completion times in the combined patient group was to a large extent driven by a significant result in the tAD group, while the PCA results were not statistically significant; this can be explained by greater variability in the completion times of the PCA patients, compared to the tAD patients. A similar pattern in the results for the two patient groups was also seen for the measure of walking path directness for the same reason. The consistent lack of evidence for overall group differences between PCA and tAD patients across primary and secondary outcomes meant we were unable to reject the subsidiary null hypothesis. However, the lack of significant differences between patient groups should be considered in the context of patient variability in completion times. Furthermore, while visual processing impairments were more apparent in PCA relative to tAD patients, they were also evident in at least one visual domain within the majority of tAD patients.

The secondary outcome results are mixed. The combined patient group showed some evidence, again driven by the tAD group, that CCue + motion increased the odds of fixating the target destination before initiating walking, supporting the hypothesized role of cues in the visual localization of targets. For the control group, the odds of fixating targets were low regardless of condition. However, the need for control participants to locate destinations through explicit fixation was likely precluded by the visibility of targets from the starting point, in combination with preserved abilities to represent the spatial layout of the setting and predict target position. For the tAD group, both cues were also weakly associated with increased directness of paths, suggesting more reliable visual localization, and walking to cued targets by these patients. However, for trials where the target was fixated during the initial period, the tAD group spent an increased proportion of time fixating cued targets, compared with non‐cued targets, before initiating walking. This appears inconsistent with tAD group improvements (with cues) for primary and other secondary outcome measures, suggesting reduced efficiency in identifying the target before starting to walk. The incongruence of cues, appearing environmentally distinct due to higher‐level, semantic rather than lower‐level perceptual factors,^35^ might require increased target processing, particularly for patients exhibiting a greater degree of memory impairment. In contrast, there was some suggestion that PCA patients who did initially fixate on the target spent a reduced proportion of time fixating before initiating movement to cued targets, supporting more efficient recognition and discrimination compared to non‐cued targets. That said, secondary outcomes are intended as only an exploration of possible mechanisms through which cues might support navigation; caution is needed when interpreting these results in isolation.

To limit bias, task instructions made no reference to the presence or absence of the cues. Future investigations could introduce cues that are information carrying through explicit instructions or appearance (e.g., directional arrows), or alternatively could use familiar cues such as personal memorabilia.^36^, ^37^ The lower visual orientation in PCA patients with particularly pronounced visual dysfunction and in the more impaired tAD patients suggests the need to investigate cues that emphasize the floorpath to a target rather than the target destination (without obstructing the floorpath^20^, ^21^). Future cues might also use audiovisual stimuli to promote target localization, with the caveat that position discrimination deficits in tAD and PCA may occur in both auditory and visual domains.^38^

To the best of our knowledge, this study represents the first empirical investigation of navigation in PCA, comprising particularly detailed assessment of perceptual factors influencing real‐world navigation in tAD and PCA. There are, however, limitations. First, the sampling frequency of the eyetracker was too low to detect saccades. Second, despite participants being removed from the experimental setting between trials, it is not possible to rule out participants fixating the setting prior to eyetracking recording. Third, for secondary outcomes, sample size was reduced by missing data, particularly for fixation measures. Fourth, caution is necessary regarding generalizability of the current study. Findings are from mostly young‐onset AD patients, who are more likely to exhibit deficits in non‐amnestic cognitive domains,^39^ and we used a simplified and unfamiliar experimental setting. Previous investigations of visual cues have outlined their benefits on wayfinding and nutrition in late‐onset AD in residential care settings.^19^, ^40^ However, without a better understanding of factors underlying patients’ navigation, it is possible that inappropriate visual cues might be detrimental in a familiar, non‐experimental setting, potentially interfering with existing salient features supporting orientation and/or increasing visual clutter.

This study provides evidence that altering the presentation of target destinations through a contrast‐based visual cue resulted in improvement to the primary outcome measure, time to destination, albeit more evidently in the tAD than PCA group. However, the addition of motion patterns did not benefit patient task performance over and above the addition of the contrast cue. Further empirical work is required to understand the influence of specific perceptual aspects of the environment on navigation, ultimately to develop aids and strategies to enhance patients’ autonomy, safety, and mobility.

## Author Contributions

K. Y.: conception and design of the study, acquisition and analysis of data, drafting a significant portion of the manuscript or figures. I. M./T. P./J. L./J. S./C. F.: acquisition and analysis of data, drafting a significant portion of the manuscript or figures. T. S./B. Y./A. C./C. H./N. P./C. S./R. P./A. F.: acquisition and analysis of data. D. B./N. T.: conception and design of the study. S. C.: conception and design of the study, drafting a significant portion of the manuscript or figures.

## Conflict of Interest

None declared.

## Supporting information


**Table S1.** Proportion of trials completed within the cut‐off time of 60 sec under baseline and cue conditions for tAD, PCA, combined patient group and controls.
