# Supplementary material for: Navigational cue effects in Alzheimer's disease and posterior cortical atrophy
Source: Ann Clin Transl Neurol. 2018 Apr 20;5(6):697–709. doi: 10.1002/acn3.566 (PMC5989777; doi:10.1002/acn3.566)
Supplement: Supplementary file 1 — Table S1. Proportion of trials completed within the cut‐off time of 60 sec under baseline and cue conditions for tAD, PCA, combined patient group and controls. [file ACN3-5-697-s001.docx]

**Table S1.** Proportion of trials completed within the cut-off time of 60 seconds under baseline and cue conditions for tAD, PCA, combined patient group and controls.

|  | **Baseline** | **CCue** | **CCue + Motion** |
| --- | --- | --- | --- |
| **tAD (N=10)** | 109/114 (95.6%) | 113/114 (99.1%) | 111/114 (97.4%) |
| **PCA (N=8)** | 74/84 (88.1%) | 76/84 (90.5%) | 74/84 (88.1%) |
| **Patients combined (N=18)** | 183/198 (92.4%) | 189/198 (95.5%) | 185/198 (93.4%) |
| **Controls (N=12)** | 144/144 (100%) | 144/144 (100%) | 144/144 (100%) |
